# Columbia Open Health Data, clinical concept prevalence and co-occurrence from electronic health records

**DOI:** 10.1038/sdata.2018.273

**Published:** 2018-11-27

**Authors:** Casey N. Ta, Michel Dumontier, George Hripcsak, Nicholas P. Tatonetti, Chunhua Weng

**Affiliations:** 1 Department of Biomedical Informatics, Columbia University, NY, USA; 2 Institute of Data Science, Maastricht University, Maastricht, The Netherlands; 3 Department of Systems Biology, Columbia University, NY, USA; 4 Department of Medicine, Columbia University, NY, USA

**Keywords:** Diseases, Diagnosis, Data mining

## Abstract

Columbia Open Health Data (COHD) is a publicly accessible database of electronic health record (EHR) prevalence and co-occurrence frequencies between conditions, drugs, procedures, and demographics. COHD was derived from Columbia University Irving Medical Center’s Observational Health Data Sciences and Informatics (OHDSI) database. The lifetime dataset, derived from all records, contains 36,578 single concepts (11,952 conditions, 12,334 drugs, and 10,816 procedures) and 32,788,901 concept pairs from 5,364,781 patients. The 5-year dataset, derived from records from 2013–2017, contains 29,964 single concepts (10,159 conditions, 10,264 drugs, and 8,270 procedures) and 15,927,195 concept pairs from 1,790,431 patients. Exclusion of rare concepts (count ≤ 10) and Poisson randomization enable data sharing by eliminating risks to patient privacy. EHR prevalences are informative of healthcare consumption rates. Analysis of co-occurrence frequencies via relative frequency analysis and observed-expected frequency ratio are informative of associations between clinical concepts, useful for biomedical research tasks such as drug repurposing and pharmacovigilance. COHD is publicly accessible through a web application-programming interface (API) and downloadable from the Figshare repository. The code is available on GitHub.

## Background & Summary

Sharing clinical data is important for reproducible biomedical research and can drive discovery. Openly available data improves the accuracy of research, enables optimal generation of knowledge, drives discoveries undetectable in individual data sets, and enhances trust in clinical research^1–3^. However, even when patient-level data are fully de-identified following the HIPAA Safe Harbor Method, patient privacy is still at risk of re-identification by using external sources of identifying information^4^. Therefore, it is important to develop methods to share clinical statistics derived from patient data without risking patient re-identification. One such method is the sharing of prevalence and co-occurrence statistics of medical events. Prevalence measures the population disease burden and can be useful to clinicians for guiding differential diagnoses, insurance providers estimating healthcare costs, pharmaceutical companies forecasting new treatment market sizes, and researchers estimating power of a clinical trial protocol^5^. Co-occurrence statistics are frequently used to determine associations between entities, such as disease-disease pairs^6^, diseases and clinical findings^7^, and adverse drug events^8^.

The prevalence, incidence rates, and other statistics of various diseases are commonly estimated from population surveys and reported in literature; however, these reports generally focus on specific classes of diseases. Grant et al. analyzed national interviews to determine the prevalence of 7 DSM-IV personality disorders and the co-occurrence among them via odds ratios^9^. Lee et al. analyzed co-occurrences of coronary artery disease, congestive heart failure, diabetes mellitus, urinary incontinence, and injurious falls within the geriatric population from health surveys^10^. The American Cancer Society releases annual reports of cancer statistics in the United States collected from cancer registries, including incidence, mortality, and survival from 46 anatomical cancer sites^11^. Although these reports can accurately estimate the disease prevalence in the general population, this knowledge is difficult to consume at scale, as it requires manual literature review. Clarivate Analytics (Philadelphia, PA) offers an Incidence and Prevalence Database including over 4000 diseases and procedures, but this database is not freely available. The National Cancer Institute provides SEER*Explorer to easily explore cancer statistics, but this database is limited to cancer.

Several research groups estimated prevalence from electronic health records (EHR) or pharmacy claims databases. Wiréhn et al. reported prevalence of diabetes, hypertension, asthma, and chronic obstructive pulmonary disease estimated from hospital and primary healthcare data in administrative databases^12^. Naughton et al. reported prevalence on 22 chronic diseases in elderly patients estimated from a pharmacy claims database^13^. Violán et al. compared prevalence estimates from health surveys vs. EHR data for 27 chronic conditions^14^. Ornstein et al. estimated prevalence and multi-morbidity of 24 chronic conditions from an EHR database covering primary care practices^15^. Bhattacharya et al. reported patterns of co-occurring conditions in patients with kidney disease by applying topic modeling on SNOMED codes^16^. Researchers who have access to an Observational Medical Outcomes Partnership (OMOP) database can use the open web applications ACHILLES and ATLAS to access useful statistics and scientific analyses, including counts per concept, prevalence rates, and frequencies of records per person^17^. Finlayson *et al.* published a data set of occurrence and co-occurrence frequencies covering ~23,000 clinical concepts (drugs, diseases, procedures, and devices) and ~18,500,000 concept pairs detected from unstructured clinical notes from 261,397 patients^18^. Finlayson et al. demonstrated how these co-occurrence frequencies are useful in many research scenarios, including computing contingency tables used in standard statistical analysis, estimating probabilities of concepts to construct Bayesian networks, and quantifying dependencies between features to improve feature selection and model design.

To accelerate translational biomedical research, we present Columbia Open Health Data (COHD), a database of EHR prevalence and co-occurrence frequencies on conditions, drugs, procedures, and demographics (sex, race, and ethnicity) observed per patient at Columbia University Irving Medical Center (CUIMC), covering 36,578 single concepts and 32,788,901 concept pairs from 5,364,781 patients. We present a novel method of collecting EHR prevalence and co-occurrence frequencies from structured EHR data in the OMOP format and sharing these statistics via a web application-programming interface (API) (http://cohd.io). Analyzing an OMOP database and sharing the code on GitHub immediately enables any institution with clinical data in OMOP format to perform this analysis and share their results. Institutions interested in joining the Observational Health Data Sciences and Informatics (OHDSI) Research Network will find an active and open community ready to help integrate new partners. These data are also available for download from the Figshare repository (Data Citation 1).

## Methods

In this article, we use the term “concept” to refer generally to clinical entities and events, such as conditions, drugs, and procedures. Concepts can vary in their level of specificity, e.g., from *Type 2 diabetes mellitus without complication* to *Metabolic disease*. We refer to clinical concepts by their concept name as defined in the OMOP Common Data Model (CDM). When concepts appear in the main body of this article, the concept name is styled in italics (e.g., *Essential hypertension* and *Chest pain*) to distinguish the formalized concepts from regular text. Similarly, we style identifier (ID) strings from the OMOP CDM in italics (e.g., *Condition*, *Drug*, and *Procedure* for domain identifiers).

**Figure 1:**
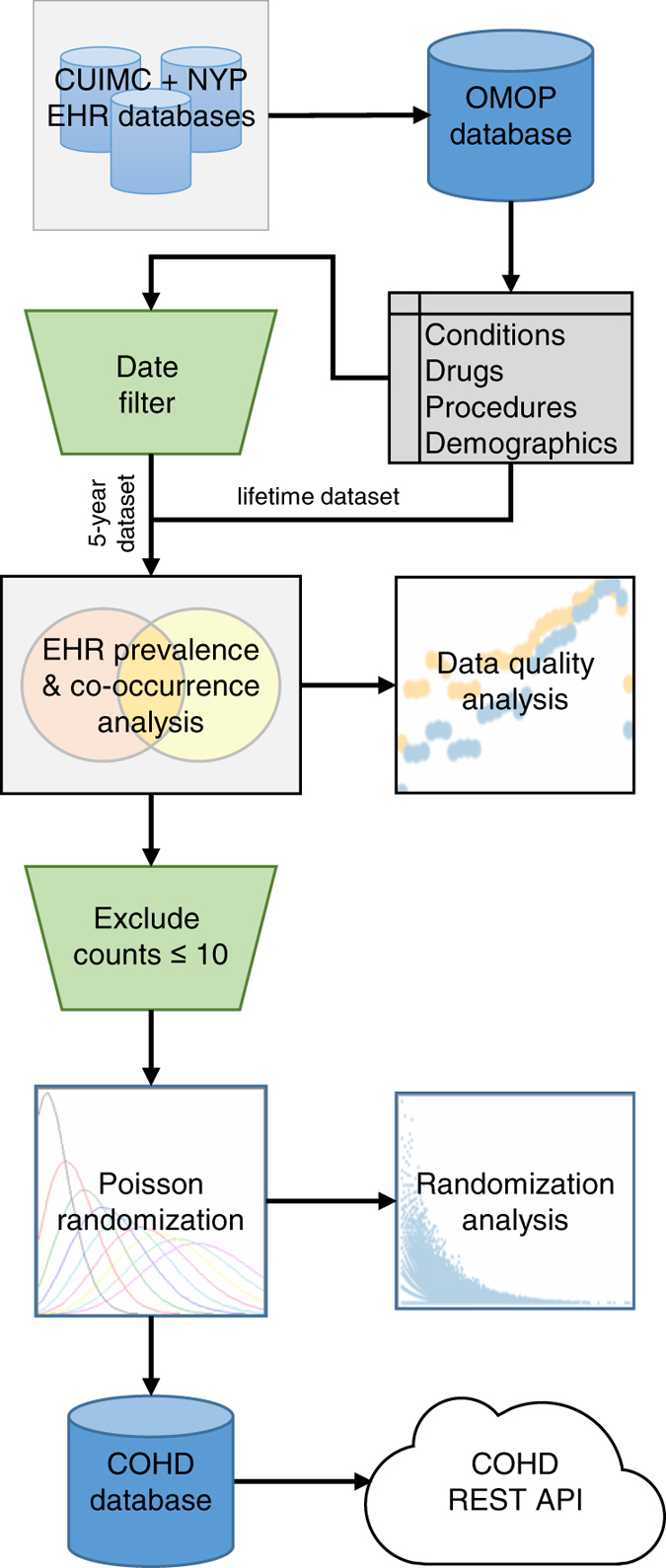
Columbia Open Health Data (COHD) workflow. Overall workflow of COHD analysis and application-programming interface (API). We analyzed an Observational Medical Outcomes Partnership (OMOP) database created from Columbia University Irving Medical Center (CUIMC) and New York Presbyterian’s (NYP) clinical data warehouse. We extracted conditions, drugs, procedures, and demographics to calculate prevalence and co-occurrence frequencies. The lifetime dataset used all data while the 5-year dataset only used data from 2013–2017. For patient protection, we excluded concepts with counts ≤ 10 and perturbed the remaining counts using Poisson randomization. The resulting data are stored in a MySQL database and served publicly via the COHD Representational State Transfer (REST) API.

Figure 1 depicts the overall workflow for generating the COHD datasets and the COHD API. Briefly, we extracted conditions, drugs, procedures, and demographics from CUIMC’s OMOP database to calculate prevalence and co-occurrence frequencies. The lifetime dataset measured prevalence and co-occurrence from data from all available years while the 5-year dataset only used data from 2013–2017. For patient protection, we excluded concepts with counts ≤ 10 and perturbed the included counts using Poisson randomization. The resulting data are stored in a MySQL database and served to the public via the COHD RESTful web API. Details of these steps follow.

### Data source

This study received institutional review board approval with waiver for informed consent. We analyzed data from CUIMC’s OHDSI database. The OHDSI database was derived from longitudinal electronic health records including inpatient and outpatient data spanning from 1985 to 2018. CUIMC’s clinical data warehouse (CDW) was converted to OMOP CDM v5.1 in March 2018. CUIMC and New York Presbyterian (NYP) Hospital serve New York, NY and the surrounding area. The diverse population of 8.2 M people in New York City, including 44.0% White, 25.5% Black, 12.7% Asian, 13.0% Some Other Race, and 4.0% Two or More Races^19^, provides an ideal environment for generating aggregated statistics.

We extracted all rows from the OMOP *condition_occurrence*, *drug_exposure*, and *procedure_occurrence* tables to provide patients’ observed conditions, drugs, and procedures using the *condition_concept_id*, *drug_concept_id*, and *procedure_concept_id* columns, respectively. We extracted patients’ sex, race, and ethnicity from the *person* table’s *gender_concept_id*, *race_concept_id*, and *ethnicity_concept_id* columns, respectively. A patient is only included in a dataset if at least one condition, drug, or procedure is observed for that patient within the dataset.

Performing this analysis on an OMOP database using OMOP standard concept identifiers provides several advantages over operating on the original CDW. OMOP is a deep information model that precisely specifies the encoding and relationship between concepts and categorizes them into domains (e.g., *Condition*, *Drug*, *Procedure*, *Gender*, *Race*, and *Ethnicity*), reducing ambiguity of the meaning and use of each concept. The OMOP standard concepts are rooted in and mapped to established vocabularies, including ICD-9-CM, SNOMED-CT, RxNorm, and UMLS, providing semantic interoperability with other knowledge sources. Performing the analysis on an OMOP database facilitates future generalizability testing and data aggregation through collaborations with other institutions in the OHDSI Research Network^17^.

### EHR prevalence and co-occurrence analyses

COHD reports the EHR prevalence and co-occurrence frequencies of concepts as detected from electronic health records. We define EHR prevalence as:

(1)PEHRC= NCNP

where $P_{\mathrm{EHR}}^{C}$is EHR prevalence of concept *C*, $N_{C}$ is the number of unique patients observed with concept *C* in a given period, and $N_{P}$ is the number of unique patients observed in the database in the same period. We define co-occurrence frequency as:

(2)COEHRC1,C2= NC1,C2NP

where ${\mathrm{CO}}_{\mathrm{EHR}}^{C_{1},C_{2}}$ is the co-occurrence frequency of concepts $C_{1}$ and $C_{2}$, and $N_{C_{1},C_{2}}$ is the number of unique patients observed with both concepts $C_{1}$ and $C_{2}$. In these analyses, $N_{C}$ is the number of patients where the specific concept ID *C* is used in the OMOP tables.

We distinguish EHR prevalence from prevalence in the general population as EHR prevalence is observational and influenced by medical care processes. For example, a hypothetical patient with condition *C* always counts towards the general population prevalence of *C*, but only contributes to $P_{\mathrm{EHR}}^{C}$ if and only if the patient has a recorded diagnosis for *C* in the medical records. We discuss the differences between EHR prevalence and general population prevalence further in the Usage Notes section.

For the patient counts, we assume person IDs in the person table uniquely identify patients. For each concept *C*, the number of unique person IDs was counted to indicate the number of patients observed with the given concept ($N_{C}$). For every pair of concepts, the number of unique patient identifiers observed with both concepts was counted to indicate the paired concept count ($N_{C_{1},C_{2}}$).

We performed the above analyses on two subsets of data. First, we analyzed the entire database without restriction by date, referred to as the lifetime dataset. Following data quality analyses, we identified a 5-year range from 2013–2017, where annual clinical data were more stable. We performed the same analyses restricted to this date range and provide the results, referred to as the 5-year dataset.

To protect patients against potential re-identification risks, any concepts with $N_{C}$ ≤ 10 or pairs of concepts with $N_{C_{1},C_{2}}$ ≤ 10 were excluded from the dataset. Furthermore, the true counts were randomized by replacing the actual count with a random draw from a Poisson distribution with the expected number of events (λ) set to the observed concept count ($\lambda =N_{C}$). The Poisson is the probabilistic distribution of events occurring in a given interval if the events occur at a known rate (λ) and independently of the time since the last event^20^. Iatrogenic concept codes were removed from the data set based on a list of 2943 potentially iatrogenic ICD-9-CM, ICD-10-CM, and SNOMED-CT concept codes (e.g., ICD-9-CM code 996.82 *Complication of transplanted liver*).

To provide a metric for assessing the temporal stability of these measurements, we calculated the mean and standard deviation of the annual prevalence and co-occurrence rates. The annual prevalence and co-occurrence rates for each dataset were calculated over the years with data for the entire year (lifetime: 1986–2017; 5-year: 2013–2017), excluding years with data for only part of the year. We randomized each year’s single concept count and co-occurrence counts as described above prior to calculating the annual mean prevalence and co-occurrence rates. We used the true counts to calculate the standard deviation.

Data resulting from these analyses are available through the COHD API and downloadable from the Figshare data repository as flat-files (Data Citation 1).

### Concept association analyses

The COHD API employs three methods to provide different perspectives on quantifying associations between concepts from co-occurrence frequencies.

### Chi-square

The most common form of association analysis from co-occurrence data is the standard chi-square analysis. The chi-square analysis is informative of the dependence between two concepts. However, this analysis becomes very sensitive with large population sizes, such that statistically significant results may not be scientifically significant.

### Relative frequency

The relative frequency indicates how frequently concept $C_{1}$ occurs among patients who have concept $C_{2}$. This is similar to the conditional probability of $C_{1}$ given $C_{2}$. Relative frequency is calculated as:

(3)FR(C1∣C2)=NC1,C2NC2

where $F_{R}(C_{1}|C_{2})$ is the relative frequency of concept $C_{1}$ among patients observed with concept $C_{2}$, $N_{C_{1},C_{2}}$ is the number of unique patients observed with both concepts $C_{1}$ and $C_{2}$, and $N_{C_{2}}$ is the count of patients with concept $C_{2}$.

### Observed-expected frequency ratio

The observed-expected frequency ratio quantifies the strength of the dependence between two concepts. The natural logarithm of observed-expected frequency ratio (log ratio for short) is calculated as:

(4)LR(C1,C2)=logeNC1,C2·NPNC1·NC2

where $\mathrm{LR}(C_{1},C_{2})$ is the log ratio of concepts $C_{1}$ and $C_{2}$, $N_{C_{1},C_{2}}$ is the number of unique patients observed with both concepts $C_{1}$ and $C_{2}$, $N_{C_{1}}$ and $N_{C_{2}}$ are the counts of patients observed with concept $C_{1}$ and concept $C_{2}$, respectively, and $N_{P}$ is the number of patients in the dataset. $\frac{N_{C_{1}\cdot }N_{C_{2}}}{N_{P}}$ estimates the expected co-occurrence count of concepts $C_{1}$ and $C_{2}$ assuming independence between the concepts. The ratio indicates whether the pair of concepts co-occurred more or less frequently relative to the expected frequency. The natural logarithm transforms the scale such that the magnitude indicates the strength of the dependence between the concepts and the sign indicates the direction.

### Code availability

The code to perform EHR prevalence and co-occurrence analyses was written in Python 2.7 and was performed on an OMOP CDM V5.1 database on Microsoft SQL Server 2014 SP2. Statistical tests were performed using the SciPy Python library version 0.19.1. The code and instructions to perform the EHR prevalence and co-occurrence analysis are publicly available on GitHub with no restrictions to access, allowing other institutions to replicate our analyses on their databases (https://github.com/CaseyTa/ehr_prevalence). The code only requires minimal modifications for any institution with an OMOP CDM v4 or v5 database.

The COHD API was implemented using FLASK (Python web framework) running on uWSGI (application server container) and Nginx (web server). The data is stored on a MySQL server running on an Amazon Relational Database Service instance. To promote and facilitate open data sharing, the code and instructions to deploy the server are publicly available with no restrictions to access on GitHub (https://github.com/CaseyTa/cohd).

## Data Records

### COHD API

The COHD API, a RESTful (Representational State Transfer) web API, provides public access to the COHD data (http://cohd.io). Table 1 lists the API endpoints and their descriptions. The API endpoints are grouped into four resources based on their functionality. The *metadata* resources provide COHD metadata, including the available datasets, the number of single concepts per domain in each data set, the number of paired concepts per domain, and the number of patients in each data set. The *OMOP* resources provide definitions of the OMOP concept IDs, a search utility to find OMOP concepts by name, endpoints that map concepts between OMOP source vocabularies and OMOP standard concepts, and endpoints that use the EMBL-EBI Ontology Xref Service (https://www.ebi.ac.uk/spot/oxo/index) to map concepts between OMOP and external ontologies. The *frequencies* resources provide access to the EHR prevalence and co-occurrence data, including endpoints to retrieve single concept counts and paired concept counts for specified concepts, lists of most frequent concepts by domain, and lists of most frequent concepts associated with a specified concept. The *association* resources provide estimates of the degree of association between concepts, including chi-square analysis, relative frequency, and observed-expected frequency ratio.

The SmartAPI page provides detailed documentation of the COHD API as well as an interactive interface that allows users to perform simple queries. The API returns data in JSON (JavaScript Object Notation) format. The use cases described below demonstrate scenarios how researchers can use the API to answer various questions. Example Python code that demonstrates how to programmatically retrieve and analyze COHD data, including these use cases, is available in a Python notebook on GitHub (https://github.com/CaseyTa/cohd/).

### Figshare

The single concept counts and paired-concept co-occurrences for the lifetime and 5-year data sets and the concept definitions are also available to download from Figshare as flat-files (Data Citation 1). Nine tab-delimited text files comprise this data record. The concept association analyses are not included in these records since they can be computed directly from single and paired-concept counts as described in the methods. In all files, concepts are referenced by their OMOP standard concept ID, and frequencies are relative to a maximum of 1.0 (1.0 = 100%).

***lifetime_single_concepts.txt***: The single concept counts from the lifetime data set. The columns are the concept ID, count of patients with this concept, and prevalence of patients with this concept.

***lifetime_paired_concepts.txt***: The paired concept counts from the lifetime data set. The columns are the first concept ID, second concept ID, count of patients with this pair of concepts, and prevalence of patients with this pair of concepts.

***lifetime_single_concept_deviations.txt***: The single concept means and standard deviations of annual prevalence from the lifetime data set. The columns are the concept ID, mean annual prevalence of this concept, and standard deviation of the annual prevalence of this concept.

***lifetime_paired_concept_deviations.txt***: The paired-concept means and standard deviations of annual co-occurrence rates from the lifetime data set. The columns are the first concept ID, second concept ID, mean annual co-occurrence rate of this concept pair, and standard deviation of the annual co-occurrence rate of this concept pair.

***5year_single_concepts.txt***: The single concept counts from the 5-year data set. The columns are the concept ID, count of patients with this concept, and prevalence of patients with this concept.

***5year_paired_concepts.txt***: The paired concept counts from the 5-year data set. The columns are the first concept ID, second concept ID, count of patients with this pair of concepts, and prevalence of patients with this pair of concepts.

***5year_single_concept_deviations.txt***: The single concept means and standard deviations of annual prevalence from the 5-year data set. The columns are the concept ID, mean annual prevalence of this concept, and standard deviation of the annual prevalence of this concept.

***5year_paired_concept_deviations.txt***: The paired-concept means and standard deviations of annual co-occurrence rates from the 5-year data set. The columns are the first concept ID, second concept ID, mean annual co-occurrence rate of this concept pair, and standard deviation of the annual co-occurrence rate of this concept pair.

***concepts.txt***: The concept definitions. The columns are the concept ID, concept name, domain, source vocabulary (the vocabulary that originally defined this concept, e.g., SNOMED, RxNorm, etc.), concept class, and concept source code (the identifier for this concept from the source vocabulary).

## Technical Validation

### EHR prevalence and co-occurrence descriptive statistics

We analyzed CUIMC’s OMOP database to detect the EHR prevalence and co-occurrence of conditions, drugs, procedures, and demographics. Table 2 lists the OMOP data tables used in this analysis along with the number of records accessed to compute the EHR prevalence and co-occurrence frequencies for the lifetime and 5-year datasets. The lifetime dataset contains count and EHR prevalence data on 36,578 single concepts and 32,788,901 pairs of concepts from 5,364,781 patients, including 11,952 conditions, 12,334 drugs, and 10,816 procedures (Table 3). The 5-year dataset contains data on 29,964 single concepts and 15,927,195 pairs of concepts from 1,790,431 patients, including 10,159 conditions, 10,264 drugs, and 8,270 procedures. Table 4 lists the counts of pairs of concepts by domain.

The EHR prevalence and co-occurrence analyses included all concepts from the OMOP *condition_occurrence*, *drug_exposure*, and *procedure_occurrence* tables (Table 2). These tables should only contain concepts from the domains *Condition*, *Drug*, and *Procedure*, respectively. However, due to errors in the extract, transform, load (ETL) process that converts CUIMC’s clinical data warehouse into the OMOP data tables, these tables also contained concepts from other domains, including *Device*, *Measurement*, *Observation*, and *Relationship* (Tables 3 and 4). Since these extraneous data do not affect the counts of concepts of interest (conditions, drugs, procedures, and demographics), we did not exclude them from the analysis. However, counts for concepts from the *Device*, *Measurement*, *Observation*, and *Relationship* domains may have limited accuracy since the relevant *device_exposure*, *measurement*, and *observation* OMOP tables were not included in the analysis.

### Data quality

To assess temporal stability of the clinical occurrence measurements, we calculated the nonrandomized counts of all concepts on a yearly basis, looking for consistency from year to year. We evaluated the yearly counts on an individual concept level for demographic data. For each of the conditions, drugs, and procedures domains, we reviewed the total counts across concepts in each domain and the count-per-capita to identify data quality issues.

**Figure 2:**
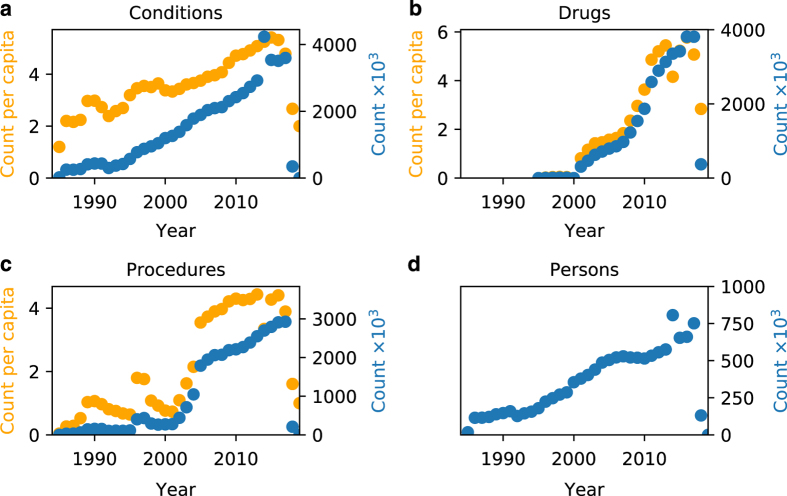
Annual total counts and counts per capita per domain. Total counts (blue) and counts per capita (orange) of **a**) condition occurrences, **b**) drug exposures, **c**) procedure occurrences, and **d**) people per year.

Figure 2 shows the total counts across all concepts (blue) and counts per capita (orange) of a) condition occurrences, b) drug exposures, c) procedure occurrences, and d) people per year. The total counts and count per capita of conditions, drugs, and people increase over time, with conditions and people beginning to increase in 1985, and drugs beginning in 2001 and increasing steadily. Speaking to an expert, we learned that the ancillary system for drugs came online in 2001 and consequently had no data for prior years. The total counts for conditions and people grow relatively steadily throughout the years, but both sets of counts spike in 2014 while the count-per-capita for conditions remains in line with its neighboring years. These data suggest that in 2014, the CUIMC database contained condition data on additional patients who were not included in other years. Counts of procedures begin in 1987 but exhibit unstable behavior prior to 2005. The total counts of procedures grew from 1987 to 1990, steadily dropped until 1995, suddenly increased in 1996-1997, dropped again until 2001, rapidly grew until 2005, and steadily grew thereafter. Counts across all domains drop in 2018 since the database only contains data for a quarter of the year.

**Figure 3:**
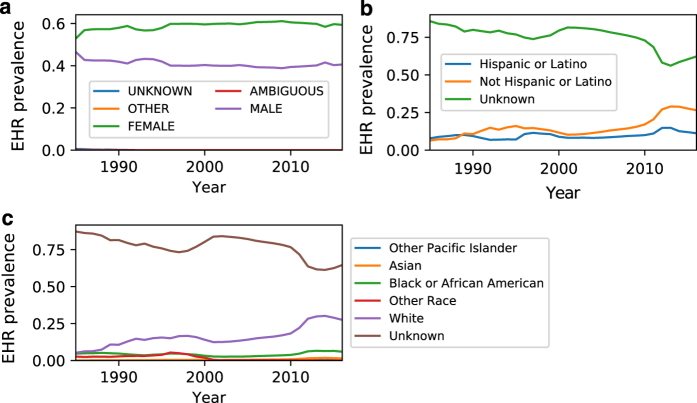
Annual demographics prevalence rates. EHR prevalence per year of **a**) sex, **b**) ethnicity, and **c**) race. **c**) For visual clarity, the plot excludes races with EHR prevalence < 0.001.

Figure 3 shows the yearly EHR prevalence of individual concepts related to a) sex, b) ethnicity, and c) race. Sex remained stable throughout the years, yielding lifetime EHR prevalence of 55.7% *FEMALE*, 44.0% *MALE*, 0.04% *AMBIGUOUS*, and 0.03% *UNKNOWN*. However, ethnicity and race data fluctuated throughout the years, with lifetime EHR prevalence of *Unknown* ethnicity and *Unknown* race accounting for 83.9% and 82.8% of the patient population, respectively. Although sex data appears reliable, missing data is a major issue for ethnicity and race. We will report an in-depth analysis of the data quality issues in a separate article.

Due to these issues, we decided to compute and provide the 5-year statistics restricted to events occurring between 2013 and 2017 in addition to the original lifetime dataset. The domain total counts within this time range are not completely stable, but they do not exhibit major fluctuations as seen prior to 2005. We make both datasets available for public consumption since certain use cases, such as detecting concept associations, may benefit from the larger sample sizes while tolerating these instabilities.

The mean and standard deviation of single concept prevalence and concept-pair co-occurrence rates calculated annually are available to assess the temporal variance of each concept and concept-pair. The mean and standard deviation of annual prevalence and co-occurrence rates should only be compared to each other to assess stability of the concept over the time period of the dataset. They should not be compared to their 5-year and lifetime counterparts since 5-year and lifetime prevalence are expected to be larger than 1-year prevalence.

### Poisson randomization

The true (nonrandomized) patient counts were perturbed using Poisson randomization to insert an additional layer of security to patient privacy. To determine the impact of the Poisson randomization on interpretation of the counts, we performed a chi-squared analysis on the counts of all single concepts and all pairs of concepts from the lifetime dataset. We compared the nonrandomized and randomized counts and calculated the frequency of statistically significant results (α = 0.05), that is, the number of concepts (or pairs of concepts) where the randomized count is significantly different from the true count divided by the total number of concepts (or pairs of concepts). This indicates how often the randomization process causes the reported counts to be significantly different from the true counts. The randomized counts were significantly different (*P* &lt; 0.05) in 4.76% (1,740/36,578) of single-concept counts and 4.93% (1,617,257/32,788,901) of paired-concept counts. This matches our expectation that when using α = 0.05, 5% of tests will reject the null-hypothesis when randomly drawn from the same sample population.

**Figure 4:**
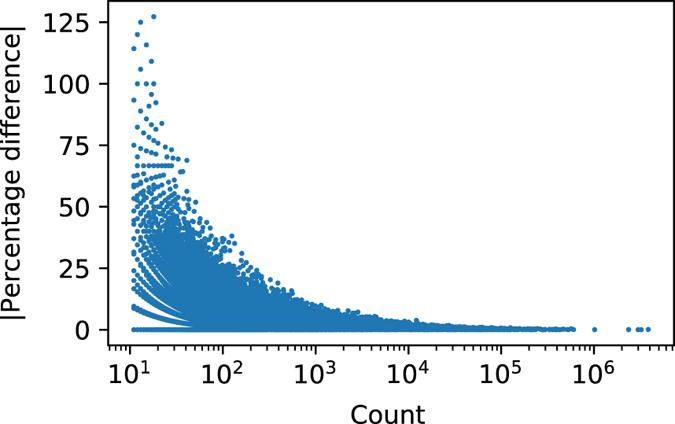
Effect of Poisson randomization. Absolute percentage difference between Poisson randomized and true counts vs true counts for single concept counts in the lifetime dataset.

To understand how the Poisson randomization affected counts at different magnitudes, we evaluated the absolute percentage difference between randomized and nonrandomized single concept counts in the lifetime dataset. Figure 4 shows a scatter plot of the absolute percentage difference vs. the true counts. The maximum absolute percentage difference decreases as the true concept count increases. This trend shows that the Poisson randomization has the largest relative effect on concepts with small counts (count &lt; 100) and small relative effect on concepts with very large counts. This is a desirable characteristic for the randomization process since rare concepts with low prevalence have greater potential risk for patient re-identification than common concepts with high prevalence. This also indicates that investigators using COHD data should be more conservative in their analyses when the count is small.

## Usage Notes

### Use case analyses

To demonstrate the utility of COHD, we discuss two general use cases for using COHD as a knowledge base or knowledge discovery platform. All results below use the 5-year dataset. To facilitate using COHD, we provide a Python notebook in our GitHub repository (https://github.com/CaseyTa/cohd) that demonstrates how to make the API calls that perform these analyses and more.

### Use case 1: knowledge base

The simplest use case for COHD is to look up the EHR prevalence of a single concept or the co-occurrence frequency of a pair of concepts. COHD references concepts by their OMOP standard concept ID. Users interested in finding out the EHR prevalence for a particular concept can search for a concept’s OMOP standard concept ID using the COHD API to perform a string similarity search. For example, an investigator interested in hypertension and hyperlipidemia will find the concept IDs 320128 (*Essential hypertension*) and 432867 (*Hyperlipidemia*), respectively. The investigator can then retrieve the EHR prevalence for *Essential hypertension* (13.1% of patients) and *Hyperlipidemia* (8.1%), and the co-occurrence frequency between the two (5.9%).

To facilitate exploration, the COHD API also provides lists of results ordered by EHR prevalence and optionally filtered by domain. For example, an investigator interested in finding out the most commonly observed conditions will find the top 10 conditions listed in Table 5. Similarly, investigators can retrieve the concepts that most commonly co-occur with a concept of interest. Table 6 lists the 10 drugs that most frequently co-occur among patients with *Atrial fibrillation*, a condition where the heart beats irregularly and often causes poor blood flow. However, analysis of the raw co-occurrence frequency between pairs of concepts may not provide much valuable insight to investigators because the most prevalent individual concepts dominate this list. For example, the drug that most commonly co-occurs with *Atrial fibrillation* is *Acetaminophen 325 MG Oral Tablet [Tylenol]*, a general pain reliever, co-occurring with *Atrial fibrillation* in 1.18% of patients. Despite not being strongly associated, *Acetaminophen 325 MG Oral Tablet [Tylenol]* likely co-occurs frequently with *Atrial fibrillation* because it has high individual prevalence (11.0%).

### Use case 2: concept associations

Instead of analyzing raw co-occurrence frequencies, sorting concepts by relative frequency (equation (3)) can yield better insight for knowledge discovery. Applying relative frequency analysis to the same question of investigating the most common drugs associated with *Atrial fibrillation* produces the results in Table 7. The relative frequency informs investigators that among patients who take the drug named in each row, this proportion of patients experience atrial fibrillation. Note that the relative frequency can exceed the upper limit of 1.0 due to the Poisson randomization. In this data, nearly all patients who take these drugs have experienced atrial fibrillation. Several of these drugs treat arrhythmia, including *dronedarone 400 MG Oral Tablet*, *Flecainide Acetate 50 MG Oral Tablet [Tambocor]*, and *Sotalol Hydrochloride 160 MG Oral Tablet [Betapace]*. Other drugs included in this list are often prescribed to treat associated conditions. For example, patients with atrial fibrillation are at risk of developing blood clots and subsequently having a stroke, thus physicians commonly prescribe anticoagulants like *dabigatran etexilate 75 MG Oral Capsule* and *Warfarin* to reduce the risk of stroke^21^.

Similarly, investigators could query COHD for conditions related to a drug of interest to find potential primary uses, off-label uses, adverse side effects, and co-occurring conditions. Table 8 shows results for this type of analysis for *Albuterol 0.83 MG/ML Inhalant Solution*, a bronchodilator commonly used in patients with asthma, bronchitis, and other lung diseases. Among these results, *Acute bronchitis due to rhinovirus* and *Acute severe exacerbation of mild persistent asthma* are the most common conditions (by paired concept count) associated with albuterol treatment and are straightforward applications for its use^22,23^. *Acute pulmonary insufficiency following thoracic surgery* and *Lung disease with systemic lupus erythematosus* are less common but still known uses for albuterol^24,25^. We could not find a documented connection between albuterol and conditions like *Gastrostomy hemorrhage* and *Leakage of cardiac device* in literature.

Additionally, the observed-expected frequency ratio (equation (4)) indicates whether a pair of concepts occur together more or less frequently than expected. Table 9 shows a sample of pairs of concepts throughout the range of association strengths. The sample pairs of concepts were automatically chosen by selecting the log ratios closest to whole values (e.g., 1, 2, 3, etc.) throughout the range. Pairs of concepts with highly positive log ratios include strongly associated concepts, such as the procedures *Extirpation of Matter from Right External Auditory Canal, Via Natural or Artificial Opening* and *Extirpation of Matter from Left External Auditory Canal, Via Natural or Artificial Opening* with a log ratio of 12.00. Pairs of concepts with log ratios near zero have weak or no association, such as *Dexamethasone phosphate 10 MG/ML Injectable Solution*, a corticosteroid used for many conditions, and *Computed tomography, cervical spine; without contrast material*, an imaging procedure. Pairs of concepts with highly negative log ratios include negatively associated concepts, such as the sex *MALE* and the procedure *Gynecologic examination* with a log ratio of −7.12. Such highly negative associations may alert to the presence of erroneous data in the EHR database when concepts that should never co-occur are recorded for the same patient.

The typical caution applies when evaluating association findings: association does not imply causality or a direct association between concepts. For example, *Benign prostatic hyperplasia* and *Gynecologic examination* have a log ratio of −4.46. However, the concepts are not directly associated, as *Benign prostatic hyperplasia* is a condition occurring in men, and *Gynecologic examination* is a procedure performed on women.

### EHR prevalence

Data consumers must understand the nuances of EHR prevalence and co-occurrence statistics when interpreting these data for their purposes. These datasets provide information about concept prevalence as recorded in the CDW. The process by which structured clinical data is entered into the CDW varies by setting. For inpatient data, after the patient is discharged, coders review patient records, including structured and narrative data, and enter diagnosis codes based on the whole record. For outpatient data, physicians, office managers, or coders enter diagnosis codes around the time of the visit. In recent years, especially after the Department of Health and Human Services’ “Meaningful Use” requirements took effect in 2010^26^, these processes ensure that most billing diagnosis codes, drug orders, and procedures are recorded in the CDW. Accordingly, COHD provides the 5-year dataset which utilizes data from 2013–2017, when structured data were reliably recorded in the CDW.

Another factor to consider when analyzing structured clinical data is the use of different clinical coding standards across time and practices. For example, the CUIMC system transitioned from using ICD-9-CM to ICD-10-CM in 2015, undergoing a formal process involving extensive training of physicians and coders. Similarly, different practices may use different coding systems, such as ICD-10-PCS, CPT4, or HCPCS to code the same concepts. The OMOP CDM alleviates most of the burden of harmonizing multiple coding systems by mapping codes from these various source vocabularies to a set of OMOP standard concepts, leveraging existing mappings in the National Library of Medicine’s Unified Medical Language System and filling gaps in the mappings as needed. For example, the concept for *atrial fibrillation* in ICD-9-CM (427.31), ICD-10-CM (I48.91), and SNOMED-CT (49436004) all map to OMOP standard concept ID 313217, rooted on the SNOMED-CT code. Issues occasionally persist due to incongruent mappings. For example, in the CUIMC CDW, the most common ICD-9-CM codes used for patients with type-2 diabetes mellitus were 250 and 250.02, which mapped to OMOP concept ID 201826 (*Type 2 diabetes mellitus*). After the transition to ICD-10-CM, E11.9 was used predominantly, which maps to the more specific OMOP concept ID 4193704 (*Type 2 diabetes mellitus without complication*).

It is tempting to interpret EHR prevalence in these datasets as lifetime prevalence and 5-year period prevalence in the general population. Although these data represent concept prevalence as recorded in the CDW, they may not accurately reflect prevalence in the general population. For example, the 5-year EHR prevalence of essential hypertension and hyperlipidemia are 13.1% and 8.1%, respectively. In contrast, the Centers for Disease Control and Prevention (CDC) reported 29.1% prevalence of hypertension in adults (age 18+) in 2011–2012^27^ and 33.5% hyperlipidemia in adults (age 20+) in 2005–2008^28^.

Several sources of bias contribute to the differences between EHR prevalence and general population prevalence. First, clinical databases contain a base population biased towards people with higher levels of existing conditions, thus biasing the measurements to overestimate the prevalence relative to the general population. Second, the nature of medical services leads to biased detection and recording of concepts. Clinicians routinely perform certain procedures, including monitoring blood pressure, thus conditions such as essential hypertension are easily detected. However, conditions not routinely examined in medical settings are under-detected. For example, dental health is not evaluated or treated in clinical settings in the United States, resulting in *Dental caries* being underreported in COHD: 0.26% EHR prevalence compared to an estimate that 92% of adults aged 20–64 have had dental caries^29^. Third, clinical codes may be absent in the EHR for a variety of reasons. Health providers may not log diagnoses codes for observed conditions when those conditions do not require treatment, diagnostic testing, or specialty referral. Similarly, patients may not seek medical treatment for their conditions or they may not mention symptoms and conditions if those symptoms seem inconsequential or unrelated to the primary concern of their visit^12,14^. Fourth, our analysis only captures information from structured EHR data and does not take advantage of the available unstructured data, e.g., progress notes, discharge summaries, radiology reports, etc. Unstructured EHR data contain a wealth of clinically significant details complementary to structured EHR data, including symptoms, diagnostic reasoning, and treatment plans. The performance of clinical applications can benefit by combining structured and unstructured data^18,30,31^.

Despite these challenges, COHD is still meaningful as a resource of clinical statistics. Although EHR prevalence may not accurately estimate general population prevalence, it may more accurately reflect healthcare consumption rates. Since the general population prevalence includes individuals who do not seek treatment for their conditions, it overestimates the number of people consuming healthcare services. In this regard, EHR prevalence may be a more useful statistic to healthcare administrators who must optimally allocate hospital resources to various services to efficiently meet demands and insurance providers estimating the costs of covering health services.

COHD may also have a strong impact as a resource for discovering and quantifying associations between clinical concepts, as demonstrated in Use Case 2. This analysis is akin to early knowledge extraction research mining journal publications or clinical narratives to detect relationships from co-occurring biomedical concepts in text^32–34^. These data have several potential real world use cases. Associations between drugs and conditions can be useful towards pharmacovigilance efforts, detecting adverse side effects from drugs when unknown positive associations occur. Conversely, when unknown negative associations occur between a concept and a condition, this association may indicate a beneficial effect of the concept on the condition. Such associations may help uncover new applications of existing drugs (drug repurposing), protective effects of genetic conditions, etc. Reasoning algorithms can consume clinical association data to make or support inferences and generate hypotheses for clinical research. The National Center for Advancing Translational Sciences (NCATS) Biomedical Data Translator (https://ncats.nih.gov/translator) is already using COHD to integrate clinical association data into knowledge graphs for reasoning and question answering.

### Limitations

There are several limitations to this work in addition to those mentioned above. We assume that each distinct person ID identifies a unique patient; however, patients may have duplicate registrations in the CDW^35^. The co-occurrence analysis does not account for temporal relations between concepts. Without restricting co-occurrences by time, we do not know whether one concept precedes the other, how closely in time they occur, or the frequency of a given concept occurring across time within a patient (i.e., chronic vs. acute). Within the lifetime dataset, there is no limit to the window of association between concepts; a hypothetical patient with a condition occurring in 1985 and a drug exposure in 2015 will still increment the co-occurrence count for the condition-drug pair. In a future study, we will restrict co-occurrence counts by various temporal relations to find associations occurring over different time windows.

The analysis for prevalence and co-occurrence rates did not take into account the hierarchical relationships between concepts, i.e., that higher-level concepts subsume lower-level concepts. For example, the count for *Ibuprofen* (concept ID 1177480) only includes entries from the CDW where the specific concept ID 1177480 was used, but does not include entries using other descendant concepts, such as *Ibuprofen 600 MG Oral Tablet* (concept ID 19019073). In a future study, we will leverage the OMOP *concept_ancestor* table to include descendant concepts in each concept’s count. This approach will also mitigate some of the issues with coding variations across time and practices as different concepts with minor semantic differences can be aggregated into higher-level concepts. Using a related approach, Hripcsak et al. evaluated nine patient phenotype cohorts, comparing gold standard cohorts queried on source ICD-9-CM and ICD-10-CM data against OMOP mapped SNOMED-CT concept sets, and observed a maximum error rate of 0.26%^36^.

To protect patient privacy, we removed all concepts with counts ≤ 10 and randomized the statistics for all concepts. These procedures remove rare concepts from the database and perturb the counts, eliminating COHD’s utility for very rare concepts and limiting confidence in statistics for infrequent concepts.

This analysis only includes a single hospital system: CUIMC and NYP. Performing the same analysis across multiple institutions will have several benefits. Aggregating this analysis across multiple sites can diversify the population, improve accuracy, increase power and sensitivity to rare conditions, and validate results by comparing across sites. We invite other institutions to join us in publicly releasing clinical statistics. We hope that this paper and the provided code serve as catalysts to encourage other institutions to share their data to the common benefit of the translational research community. In future studies, we plan to collaborate with other institutions to compare EHR prevalence and co-occurrence frequencies across sites to assess agreement, generalizability, variance, and concept coverage across sites. We also plan to take a deeper investigation into the concept associations to validate the association detection and evaluate their utility for reasoning tasks and hypothesis generation.

## Additional information

**How to cite this article**: Ta, C. N. *et al*. Columbia Open Health Data, clinical concept prevalence and co-occurrence from electronic health records. *Sci. Data*. 5:180273 doi: 10.1038/sdata.2018.273 (2018).

**Publisher’s note**: Springer Nature remains neutral with regard to jurisdictional claims in published maps and institutional affiliations.

## Supplementary Material



## Figures and Tables

**Table 1:** COHD application-programming interface (API) endpoints.

API endpoint	Description
/metadata/datasets	Enumerates the datasets available in COHD
/metadata/domainCounts	The number of concepts in each domain
/metadata/domainPairCounts	The number of pairs of concepts in each pair of domains
/metadata/patientCount	The number of patients in the dataset
/omop/findConceptIDs	Search for OMOP concepts by name and domain
/omop/concepts	Concept definitions from concept ID
/omop/vocabularies	List of vocabularies
/omop/mapFromStandardConceptID	Map from a standard concept ID to concept code(s) in an external vocabulary
/omop/mapToStandardConceptID	Map from a non-standard concept code to a standard OMOP concept ID
/omop/xrefFromOMOP	Cross-reference from an ontology to OMOP standard concepts using the Ontology Xref Service
/omop/xrefToOMOP	Cross-reference from an ontology to OMOP standard concepts using the Ontology Xref Service
/frequencies/singleConceptFreq	Clinical frequency of individual concepts
/frequencies/pairedConceptFreq	Clinical frequency of a pair of concepts
/frequencies/mostFrequencyConcepts	Most frequent concepts [optional: by domain]
/frequencies/associatedConceptFreq	Clinical frequencies of all pairs of concepts given a concept id
/frequencies/associatedConceptDomainFreq	Clinical frequencies of all pairs of concepts given a concept id
/association/chiSquare	Chi-square analysis of paired concepts
/association/obsExpRatio	Observed Count / Expected Count
/association/relativeFrequency	Relative frequency between pairs of concepts
The COHD API endpoints are listed along with a brief description of each endpoint. The endpoints are grouped into four resources based on function: metadata, OMOP, frequencies, and association.	

**Table 2:** Number of EHR records evaluated.

OMOP CDM table name	Number of records (Lifetime)	Number of records (5-year)
condition_occurrence	140,300,457	60,057,858
drug_exposure	78,878,159	45,298,565
procedure_occurrence	64,383,775	26,030,193
person	5,364,781	1,790,431
Number of records in the Columbia Observational Medical Outcomes Partnership (OMOP) Common Data Model (CDM) v5 tables as of March 2018 used to generate the lifetime and 5-year datasets.		

**Table 3:** Number of concepts by domain for single concept counts.

Domain	Lifetime Dataset				5-Year Dataset			
	Count	Min Prevalence^1^	Mean Prevalence^1^	Max Prevalence^1^	Count	Min Prevalence^1^	Mean Prevalence^1^	Max Prevalence^1^
Condition	11952	5.59E-07	5.68E-04	8.57E-02	10159	2.00E-06	7.58E-04	1.31E-01
Device	204	1.491E-06	1.10E-04	6.19E-03	170	3.00E-06	2.15E-04	9.76E-03
Drug	12334	5.59E-07	3.82E-04	7.10E-02	10264	2.00E-06	7.71E-04	1.10E-01
Ethnicity	2	5.55E-02	8.05E-02	1.05E-01	2	1.09E-01	1.65E-01	2.22E-01
Gender	4	2.88E-04	2.50E-01	5.57E-01	4	6.00E-06	2.50E-01	5.79E-01
Measurement	235	1.68E-06	1.71E-03	8.14E-02	188	3.00E-06	3.67E-03	1.28E-01
Observation	993	9.32E-07	1.50E-03	7.18E-01	870	3.00E-06	2.80E-03	6.64E-01
Procedure	10816	5.592E-07	5.14E-04	6.02E-01	8270	2.00E-06	7.47E-04	2.41E-01
Race	32	1.491E-06	7.68E-03	1.12E-01	32	6.00E-06	1.04E-02	2.34E-01
Relationship	6	2.05E-06	1.80E-05	8.60E-05	5	1.20E-05	5.90E-05	2.33E-04
^1^Data are the minimum, mean, and maximum prevalence across concepts within the domain, respectively. Prevalence = number of patients with the concept/the total number of patients in the dataset.								
This table lists the number of unique concepts in each domain for the resulting lifetime and 5-year datasets. Descriptive statistics include the minimum, mean, and maximum prevalence among the concepts in each domain.								

**Table 4:** Number of pairs of concepts by domain for paired concept counts.

Domain 1	Domain 2	Lifetime Dataset				5-Year Dataset			
		Count	Min Prevalence^1^	Mean Prevalence^1^	Max Prevalence^1^	Count	Min Prevalence^1^	Mean Prevalence^1^	Max Prevalence^1^
Condition	Condition	4558391	0.00E + 00	3.00E-05	3.02E-02	1933917	0.00E + 00	5.40E-05	5.89E-02
Condition	Device	51651	1.86E-07	1.90E-05	4.88E-03	20045	5.59E-07	3.50E-05	7.11E-03
Condition	Drug	8384345	0.00E + 00	2.80E-05	3.16E-02	4139944	0.00E + 00	5.80E-05	4.28E-02
Condition	Ethnicity	17135	3.73E-07	1.40E-04	2.01E-02	14297	1.12E-06	2.62E-04	4.21E-02
Condition	Gender	20438	3.73E-07	3.32E-04	4.55E-02	16641	1.68E-06	4.62E-04	6.85E-02
Condition	Measurement	282893	0.00E + 00	4.40E-05	3.90E-02	168514	5.59E-07	8.50E-05	5.73E-02
Condition	Observation	725341	0.00E + 00	4.30E-05	5.39E-02	442601	0.00E + 00	9.60E-05	6.86E-02
Condition	Procedure	5359461	0.00E + 00	3.60E-05	6.67E-02	2252358	0.00E + 00	7.00E-05	5.49E-02
Condition	Race	29851	1.86E-07	7.90E-05	2.07E-02	21300	5.59E-07	1.67E-04	4.36E-02
Condition	Relationship	134	1.49E-06	5.00E-06	2.90E-05	115	2.79E-06	1.40E-05	8.50E-05
Device	Device	588	9.32E-07	1.40E-05	2.30E-03	383	1.12E-06	3.00E-05	1.03E-03
Device	Drug	45589	0.00E + 00	2.00E-05	2.29E-03	21921	1.68E-06	3.90E-05	4.51E-03
Device	Ethnicity	263	7.46E-07	4.10E-05	1.92E-03	224	2.79E-06	8.70E-05	3.45E-03
Device	Gender	343	9.32E-07	6.60E-05	3.39E-03	284	3.91E-06	1.27E-04	5.46E-03
Device	Measurement	1881	5.59E-07	3.00E-05	3.80E-03	929	1.68E-06	7.10E-05	8.18E-03
Device	Observation	5672	3.73E-07	2.60E-05	3.99E-03	3465	1.12E-06	6.30E-05	7.32E-03
Device	Procedure	35631	3.73E-07	2.20E-05	5.74E-03	14781	1.12E-06	4.20E-05	9.63E-03
Device	Race	316	7.46E-07	3.40E-05	2.04E-03	252	1.68E-06	8.20E-05	3.59E-03
Drug	Drug	4465396	0.00E + 00	3.00E-05	4.23E-02	2441654	0.00E + 00	6.90E-05	5.74E-02
Drug	Ethnicity	16984	1.86E-07	1.25E-04	1.66E-02	14079	1.12E-06	2.88E-04	3.11E-02
Drug	Gender	20558	3.73E-07	2.29E-04	4.27E-02	16929	1.12E-06	4.67E-04	6.83E-02
Drug	Measurement	278313	1.86E-07	4.20E-05	4.14E-02	178208	5.59E-07	9.40E-05	5.98E-02
Drug	Observation	712097	0.00E + 00	4.10E-05	4.51E-02	474720	0.00E + 00	1.04E-04	5.92E-02
Drug	Procedure	5223373	0.00E + 00	3.40E-05	5.88E-02	2520639	0.00E + 00	7.40E-05	5.31E-02
Drug	Race	26680	3.73E-07	7.40E-05	1.81E-02	21492	1.12E-06	1.83E-04	3.52E-02
Drug	Relationship	132	9.32E-07	4.00E-06	2.10E-05	104	3.91E-06	1.30E-05	6.10E-05
Ethnicity	Gender	8	2.24E-06	2.02E-02	5.92E-02	5	1.90E-05	6.62E-02	1.27E-01
Ethnicity	Measurement	382	1.12E-06	3.88E-04	1.74E-02	320	5.59E-06	9.54E-04	3.24E-02
Ethnicity	Observation	1305	1.12E-06	3.19E-04	2.84E-02	1169	2.79E-06	8.82E-04	8.44E-02
Ethnicity	Procedure	13121	3.73E-07	1.54E-04	7.32E-02	9751	1.12E-06	3.18E-04	6.60E-02
Ethnicity	Race	44	2.24E-06	2.87E-03	6.66E-02	43	3.91E-06	5.88E-03	1.47E-01
Ethnicity	Relationship	3	2.42E-06	8.00E-06	1.90E-05	3	9.49E-06	2.40E-05	5.20E-05
Gender	Measurement	407	1.12E-06	9.88E-04	4.30E-02	328	3.35E-06	2.10E-03	6.85E-02
Gender	Observation	1613	7.46E-07	9.23E-04	4.05E-01	1400	2.23E-06	1.74E-03	3.85E-01
Gender	Procedure	17049	5.59E-07	3.25E-04	3.47E-01	12159	5.59E-07	5.07E-04	1.43E-01
Gender	Race	58	1.68E-06	4.23E-03	6.17E-02	51	3.91E-06	6.52E-03	1.31E-01
Gender	Relationship	8	7.46E-07	1.20E-05	5.50E-05	8	4.47E-06	3.80E-05	1.93E-04
Measurement	Measurement	5421	3.73E-07	1.06E-04	1.18E-02	3922	2.23E-06	2.51E-04	3.52E-02
Measurement	Observation	24619	3.73E-07	8.00E-05	5.40E-02	17901	1.68E-06	1.98E-04	7.50E-02
Measurement	Procedure	174390	1.86E-07	6.00E-05	6.45E-02	97220	5.59E-07	1.28E-04	6.46E-02
Measurement	Race	901	9.32E-07	1.60E-04	1.90E-02	770	2.23E-06	3.95E-04	3.61E-02
Measurement	Relationship	15	1.49E-06	9.00E-06	5.40E-05	12	3.35E-06	3.20E-05	1.51E-04
Observation	Observation	34883	3.73E-07	7.90E-05	3.81E-02	26658	1.12E-06	2.25E-04	1.00E-01
Observation	Procedure	466832	1.86E-07	5.40E-05	4.53E-01	275586	5.59E-07	1.24E-04	1.84E-01
Observation	Race	2383	7.46E-07	1.61E-04	2.74E-02	2123	1.68E-06	4.55E-04	8.09E-02
Observation	Relationship	57	1.30E-06	1.30E-05	6.90E-05	55	3.35E-06	4.00E-05	1.98E-04
Procedure	Procedure	1761248	0.00E + 00	4.30E-05	1.36E-01	743843	0.00E + 00	8.70E-05	8.70E-02
Procedure	Race	20606	5.59E-07	9.40E-05	7.81E-02	14001	1.68E-06	2.13E-04	6.68E-02
Procedure	Relationship	89	1.12E-06	8.00E-06	7.60E-05	68	3.91E-06	2.70E-05	2.18E-04
Race	Relationship	3	2.24E-06	1.10E-05	2.90E-05	3	6.70E-06	3.60E-05	8.80E-05
^1^Data are the minimum, mean, and maximum prevalence across concepts within the pair of domains, respectively. Prevalence = number of patients with the concept/the total number of patients in the dataset.									
This table lists the number of unique pairs of concepts in each pair of domains for the resulting lifetime and 5-year datasets. Descriptive statistics include the minimum, mean, and maximum co-occurrence prevalence among the concept pairs in each domain.									

**Table 5:** Most prevalent conditions.

concept	count^1^	EHR prevalence^2^
Essential hypertension	233790	0.130577
Chest pain	152005	0.084899
Hyperlipidemia	145367	0.081191
Abdominal pain	124820	0.069715
Dyspnea	103718	0.057929
Inflamed seborrheic keratosis	96902	0.054122
Cough	94453	0.052754
Neoplasm of uncertain behavior of skin	85041	0.047498
Coronary arteriosclerosis in native artery	80244	0.044818
Electrocardiogram abnormal	75524	0.042182
^1^Data are the number of patients with the specified condition from the 5-year dataset.		
^2^Data are the EHR prevalence of the specified condition from the 5-year dataset. EHR prevalence = count/patient population; patient population is 1,790,431.		
The top 10 conditions with the highest EHR prevalence are listed.		

**Table 6:** Top drugs co-occurring with atrial fibrillation.

concept	count^1^	EHR prevalence^2^
Acetaminophen 325 MG Oral Tablet [Tylenol]	21165	0.011821
0.5 ML pneumococcal capsular polysaccharide type 1 vaccine 0.05 MG/ML/pneumococcal capsular polysaccharide type 10 A vaccine 0.05 MG/ML/pneumococcal capsular polysaccharide type 11 A vaccine 0.05 MG/ML/pneumococcal capsular polysaccharide type 12 F vac^3^	18340	0.010243
Docusate Sodium 100 MG Oral Capsule	17130	0.009568
1000 ML Sodium Chloride 9 MG/ML Injection	16804	0.009385
sennosides, USP 8.6 MG Oral Tablet	15877	0.008868
Aspirin 81 MG Delayed Release Oral Tablet	15554	0.008687
heparin sodium, porcine 5000 UNT/ML Injectable Solution	15215	0.008498
Aspirin 81 MG Oral Tablet	13716	0.007661
2 ML Ondansetron 2 MG/ML Injection	13526	0.007555
Acetaminophen 325 MG/Oxycodone Hydrochloride 5 MG Oral Tablet	13041	0.007284
^1^Data are the number of patients with the specified condition from the 5-year dataset.		
^2^Data are the EHR prevalence of the specified condition from the 5-year dataset. EHR prevalence = count/patient population; patient population is 1,790,431.		
^3^The name of this concept was truncated due to the 255 character limit for concept names in the OMOP CDM.		
The top ten drugs with the highest co-occurrence count with atrial fibrillation are listed.		

**Table 7:** Drugs with the highest relative frequency among patients with atrial fibrillation.

concept	paired concept count^1^	base concept count^2^	relative frequency^3^
50 ML idarucizumab 50 MG/ML Injection [Praxbind]	15	11	1.364
dronedarone 400 MG Oral Tablet	45	37	1.216
dabigatran etexilate 75 MG Oral Capsule	39	34	1.147
5 ML Dopamine Hydrochloride 80 MG/ML Injection	17	15	1.133
Flecainide Acetate 50 MG Oral Tablet [Tambocor]	16	15	1.067
darbepoetin alfa 0.025 MG/ML Injection	24	23	1.043
ovine digoxin immune fab 40 MG Injection [DigiFab]	31	31	1.000
Diltiazem Hydrochloride 90 MG Oral Tablet [Cardizem]	58	58	1.000
Warfarin	51	53	0.962
Sotalol Hydrochloride 160 MG Oral Tablet [Betapace]	42	44	0.955
^1^Data are the number of patients exposed to the specified drug and atrial fibrillation from the 5-year dataset.			
^2^Data are the number of patients exposed to the specified drug in the 5-year dataset.			
^3^Data are the ratio [paired concept count]/[base concept count] (equation (3)). Relative frequency can exceed the upper limit of 1.0 due to Poisson randomization.			
The top ten drugs associated with atrial fibrillation via relative frequency analysis are listed.			

**Table 8:** Conditions with the highest relative frequency among patients taking albuterol.

concept	paired concept count^1^	base concept count^2^	relative frequency^3^
Gastrostomy hemorrhage	16	13	1.23076923
Zygomycosis	21	18	1.166666666
Lung disease with systemic lupus erythematosus	18	16	1.125
Acute severe exacerbation of intrinsic asthma	37	33	1.121212121
Acute bronchitis due to rhinovirus	100	90	1.111111111
Acute pulmonary insufficiency following thoracic surgery	41	37	1.108108108
Injury of retroperitoneum without open wound into abdominal cavity	13	12	1.083333333
Leakage of cardiac device	18	17	1.058823529
Tracheostomy hemorrhage	19	18	1.055555555
Acute severe exacerbation of mild persistent asthma	191	183	1.043715846
^1^Data are the number of patients observed with the specified condition and albuterol from the 5-year dataset.			
^2^Data are the number of patients observed with the specified condition in the 5-year dataset.			
^3^Data are the ratio [paired concept count]/[base concept count] (equation (3)). Relative frequency can exceed the upper limit of 1.0 due to Poisson randomization.			
The top 10 conditions associated with albuterol via relative frequency analysis are listed.			

**Table 9:** Sample associated concept pairs via observed-expected frequency ratio analysis.

concept 1	concept 2	count^1^	log ratio^2^
Extirpation of Matter from Right External Auditory Canal, Via Natural or Artificial Opening	Extirpation of Matter from Left External Auditory Canal, Via Natural or Artificial Opening	19	12.00
Unilateral repair of femoral hernia with graft or prosthesis	Obstructed femoral hernia	18	11.00
Treprostinil 5 MG/ML Injectable Solution [Remodulin]	Treprostinil 10 MG/ML Injectable Solution	20	10.00
Construction of tracheoesophageal fistula and subsequent insertion of an alaryngeal speech prosthesis (eg, voice button, Blom-Singer prosthesis)	Cervical lymphadenectomy (modified radical neck dissection)	18	9.00
Repair of pulmonary venous stenosis	Total anomalous pulmonary venous return	15	8.00
Desmopressin Acetate 0.01 MG/ACTUAT Nasal Spray	Panhypopituitarism	57	7.00
Arthrodesis, posterior technique, craniocervical (occiput-C2)	Short-latency somatosensory evoked potential study, stimulation of any/all peripheral nerves or skin sites, recording from the central nervous system; in upper and lower limbs	15	6.00
Insertion of Infusion Device into Right Atrium, Percutaneous Approach	Neutropenia	60	5.00
Other partial resection of small intestine	Post-traumatic wound infection	9	4.00
carvedilol 3.125 MG Oral Tablet [Coreg]	BCR/ABL1 (t(9;22)) (eg, chronic myelogenous leukemia) translocation analysis; minor breakpoint, qualitative or quantitative	18	3.00
Fine needle aspiration; with imaging guidance	B-cell lymphoma	10	2.00
Electrocardiogram, routine ECG with at least 12 leads; interpretation and report only	Contusion	1238	1.00
Dexamethasone phosphate 10 MG/ML Injectable Solution	Computed tomography, cervical spine; without contrast material	22	0.00
Arthropathy of knee joint	Hemorrhage in early pregnancy, antepartum	11	-1.00
Postmature infancy	Human papilloma virus screening	29	-2.00
Infectious agent detection by nucleic acid (DNA or RNA); Neisseria gonorrhoeae, amplified probe technique	Technetium tc-99m sestamibi, diagnostic, per study dose	17	-3.00
Level IV - Surgical pathology, gross and microscopic examination Abortion - spontaneous/missed Artery, biopsy Bone marrow, biopsy Bone exostosis Brain/meninges, other than for tumor resection Breast, biopsy, not requiring microscopic evaluation of surgica	Hearing Screening Assessment	39	-4.00
Benign prostatic hyperplasia	FEMALE	75	-4.98
Lower urinary tract symptoms	FEMALE	12	-6.01
Gynecologic examination	MALE	56	-7.12
^1^Data are the number of patients with both concepts.			
^2^Data are calculated as log(observed paired concept count/expected paired concept count) (equation (4)).			
The sample pairs of concepts were automatically chosen by selecting concept pairs with log ratios closest to whole values (e.g., 1, 2, 3, etc.) throughout the range of log ratios.			
